# Developing and maintaining health emergency response capacity: Palau’s national emergency medical team

**DOI:** 10.5365/wpsar.2023.14.6.1039

**Published:** 2023-12-19

**Authors:** May Morag Ferguson, Sean T Casey, Wally Omengkar, Gaafar J Uherbelau, Terepkul Ngiraingas, Belinda Eungel

**Affiliations:** a World Health Organization Country Liaison Office for the Federated States of Micronesia, Marshall Islands and Palau, Palikir, Pohnpei, Federated States of Micronesia.; b World Health Organization Regional Office for the Western Pacific, Manila, Philippines.; c School of Population Health, Medicine and Health, University of New South Wales, Sydney, New South Wales, Australia.; d Ministry of Health and Human Services, Koror, Palau.; e Belau National Hospital, Koror, Palau.

The Republic of Palau is a small island–large ocean nation in the western Pacific ocean. A national census conducted in 2020 determined the population to be 17 614. (*1*) The nation is extremely susceptible to the impacts of climate change and natural hazards. (*2*) In June 2022, Palau established a national emergency medical team (EMT), making it the smallest country by population to achieve this. The team was given the name KLEMAT, a word that refers to the rope that holds the sails of Palau’s traditional canoes and also describes leadership, authority and good governance. (*3*)

KLEMAT was developed through a multistep process with support from technical and funding partners. As part of the Global Emergency Medical Team Initiative, (*4*) Palau was introduced to EMTs in 2019 through orientation webinars and virtual meetings led by the World Health Organization (WHO). These sessions were attended by key personnel from the Palau Ministry of Health and Human Services from 2019 to 2021. In 2022, WHO and the Ministry jointly hired a national EMT coordinator, and in April 2022, a Technical Working Group was formed comprising stakeholders with key roles in developing national responses to disasters (**Fig. 1**).

**Fig. 1:**
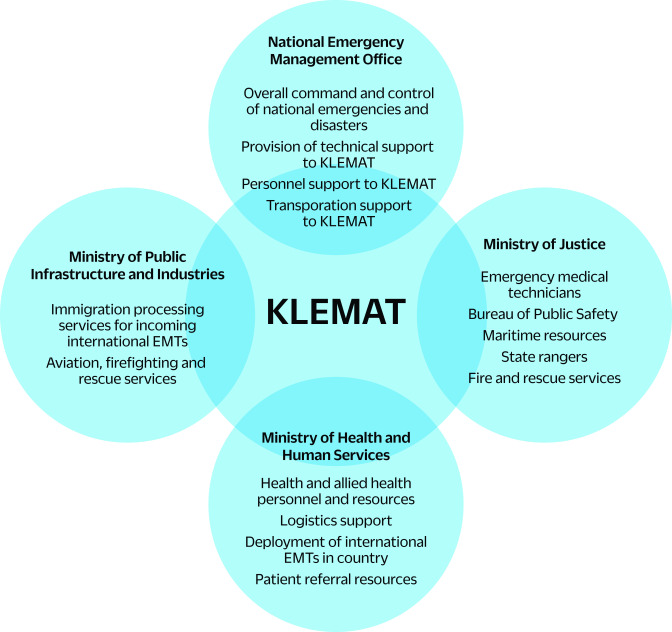
Technical Working Group comprising key stakeholders and their resource commitments to Palau’s emergency medical team (KLEMAT), 2022

[**insert** Figure 1]

The main goal of the Technical Working Group was to facilitate the establishment of a national EMT capable of being self-sufficient and providing high-quality clinical responses to emergencies or disasters. The Technical Working Group is now working towards integrating KLEMAT into the National Risk Disaster Management Framework, (*2*) the Belau National Hospital Emergency Plan and the national budget.

## Team development

As a result of the initial meetings of the Technical Working Group, KLEMAT’s standard operating procedures (SOPs) were drafted, by adapting WHO’s *Classification and minimum standards for emergency medical teams* to a small island context. (*5*) KLEMAT’s first EMT member training and full-scale simulation exercise were conducted in Palau by WHO-led faculty in June 2022. (*6*) In 2023, the 18 fully trained KLEMAT members include physicians, nurses, environmental health officers and logisticians. The number of KLEMAT personnel will continue to increase, as a result of additional WHO-led EMT member training. This expansion of KLEMAT’s roster will ensure that it has a full complement of personnel for any major deployment (**Table 1**).

**Table 1:** Palau emergency medical team (KLEMAT) personnel and roles, 2022

Role	Specialty	Description	No. of personnel
Medical director	Doctor	Overall responsibility for the Health Emergency Operations Centre and in charge of KLEMAT deployment	1
Focal point coordinator	Coordinator	Manages the roster, mobilizes the team, oversees pre-deployment preparations, works with the Health Emergency Operations Centre during deployment, oversees demobilization, acts as single source for emergency contacts	1
Administrator	Team administration, human resources	Manages patient documentation, team administration	1–2
National Emergency Management Office	Operational support	Provides advice, agreed resources and on-site support to KLEMAT	1–2
Team leader	KLEMAT team leader	Manages the team, makes decisions during deployment, acts as point of contact for any agencies involved, monitors wellness of team	1
Emergency medical technician	Triage	Manages triage on site and manages security if required; maintains ambulance equipment if applicable	2–4
Clinical lead	Doctor	Oversees all patient care: acts as resuscitation team leader, provides initial care for patients requiring emergency intervention, performs minor surgeries, acts as obstetric care lead	1–3
Nurse	Clinical	Manages all patient care in cooperation with the doctor, manages infection control in cooperation with IPC staff	1–4
Nurse specialist	IPC	Acts as forward deployment to assess site and patient expectations, relays information back to base, manages and advises all staff regarding infection control, supervises isolation tent if applicable	1–2
Logistician	Logistics	Manages storage, maintenance, accountability and distribution of cache; prepares cache for EMT to pick up; accompanies EMT if required	1–4
Environmental health	WASH	Acts as forward deployment to prepare for WASH, manages environmental health issues and vector control, advises on waste management	1–4
Active and support services	Palau Red Cross	Dependent on skill set	1–4
Pharmacist	Pharmacy management (remains at Belau National Hospital)	Orders, prepares and maintains medications for EMT use; provides medication labels and dispensing packs for EMT	1
Maintenance	Maintenance specialist (remains at Belau National Hospital unless required on site)	Performs maintenance on all equipment, electronics, generator, and lighting, both while stored and on site, as required	1

EMT: emergency medical team; IPC: infection prevention and control; WASH: water, sanitation and hygiene.

*Source:* KLEMAT Standard Operating Procedures (unpublished).

To enable KLEMAT’s activation and a fully self-sufficient response to emergencies or disasters, even in remote or austere contexts, a specifically designed EMT cache (i.e. equipment) was procured for all Pacific EMTs, including KLEMAT. (*7*) This includes personal deployment, clinical and communication equipment, among others.

Because KLEMAT has developed SOPs, been trained and has a deployment-ready cache, the team is now a deployable clinical resource, ready to respond locally, nationally and potentially regionally in the event of an emergency or disaster; KLEMAT is capable of providing fully self-sufficient tented outpatient and emergency medical services in the most challenging circumstances.

## Continuation training and motivational impetus

Post-training, the challenge for the KLEMAT team was maintaining members’ motivation and skills and continuing to develop the team’s deployment preparedness. This involved three key elements: goal-setting, establishing ground rules and clarifying roles. (*8*) KLEMAT team members were involved in establishing their own SOPs, which in turn addressed those three key elements. The challenge was then to consider how to further develop the team while maintaining their motivation. Salas et al. highlight that developing teams further can be achieved only by providing ongoing support, education and skills training. (*9*) To address this, the team began holding monthly training sessions or meetings. At each meeting, team members are asked if they have any specific training requests, thus enabling the facilitators to develop training to meet the team’s needs.

These sessions have been deliberately varied and have used local and international services and experts. The sessions have included:

casualty simulation exercises;
radio and verbal communications;
table-top exercises, covering topics such as optimal site setup for responses and local disaster scenarios;
visits to tertiary response units to increase KLEMAT’s awareness of possible external resources and to explore the capacity and capability of these units in the event of tertiary assistance being requested for Palau during a major disaster; and
external specialist speakers.

This approach to ongoing education and skills practice is supported by research that confirms that continuing training, such as simulation-based training, can be highly effective if correctly matched with the team’s skill set. (*10*) In addition to regular team training, international specialized training that is relevant to the team’s skills has been arranged. In May 2023, the Australian Defence Force sponsored two KLEMAT members, one logistician and one water, sanitation and hygiene (or WASH) specialist, to attend short courses in Australia.

Maintaining motivation among team members has been further achieved by providing activities that are varied, relevant and essential for building on existing skill sets, hence assisting the team’s overall readiness to respond effectively to any emergency or disaster. In addition, and as a result of the ongoing training, the team continues to develop as a cohesive group and members are readily able to step into their roles, as observed by training facilitators and Palau’s national EMT coordinator during training exercises.

## Conclusions

In the future, KLEMAT will invite regional EMTs to join its training sessions. This initiative will provide KLEMAT with external options for learning as well as expand the regional EMT support network. Palau’s development and ongoing training of its national EMT demonstrates that even the smallest of countries can establish a self-sufficient and motivated team capable of national and regional responses.
